# Quantifying Myocardial Blood Flow and Resistance Using 4D-Flow Cardiac Magnetic Resonance Imaging

**DOI:** 10.1155/2023/3875924

**Published:** 2023-02-02

**Authors:** Rebecca C. Gosling, Gareth Williams, Abdulaziz Al Baraikan, Samer Alabed, Eylem Levelt, Amrit Chowdhary, Peter P. Swoboda, Ian Halliday, D. Rodney Hose, Julian P. Gunn, John P. Greenwood, Sven Plein, Andrew J. Swift, James M. Wild, Pankaj Garg, Paul D. Morris

**Affiliations:** ^1^Department of Infection Immunity and Cardiovascular Disease, University of Sheffield, Sheffield, UK; ^2^Department of Cardiology, Sheffield Teaching Hospitals NHS Foundation Trust, Sheffield, UK; ^3^Insigneo Institute for in Silico Medicine, Sheffield, UK; ^4^Leeds Institute of Cardiovascular and Metabolic Medicine, University of Leeds, Leeds, UK; ^5^Norwich Medical School, University of East Anglia, Norwich, UK

## Abstract

**Background:**

Ischaemia with nonobstructive coronary arteries is most commonly caused by coronary microvascular dysfunction but remains difficult to diagnose without invasive testing. Myocardial blood flow (MBF) can be quantified noninvasively on stress perfusion cardiac magnetic resonance (CMR) or positron emission tomography but neither is routinely used in clinical practice due to practical and technical constraints. Quantification of coronary sinus (CS) flow may represent a simpler method for CMR MBF quantification. 4D flow CMR offers comprehensive intracardiac and transvalvular flow quantification. However, it is feasibility to quantify MBF remains unknown.

**Methods:**

Patients with acute myocardial infarction (MI) and healthy volunteers underwent CMR. The CS contours were traced from the 2-chamber view. A reformatted phase contrast plane was generated through the CS, and flow was quantified using 4D flow CMR over the cardiac cycle and normalised for myocardial mass. MBF and resistance (MyoR) was determined in ten healthy volunteers, ten patients with myocardial infarction (MI) without microvascular obstruction (MVO), and ten with known MVO.

**Results:**

MBF was quantified in all 30 subjects. MBF was highest in healthy controls (123.8 ± 48.4 mL/min), significantly lower in those with MI (85.7 ± 30.5 mL/min), and even lower in those with MI and MVO (67.9 ± 29.2 mL/min/) (*P* < 0.01 for both differences). Compared with healthy controls, MyoR was higher in those with MI and even higher in those with MI and MVO (0.79 (±0.35) versus 1.10 (±0.50) versus 1.50 (±0.69), *P*=0.02).

**Conclusions:**

MBF and MyoR can be quantified from 4D flow CMR. Resting MBF was reduced in patients with MI and MVO.

## 1. Introduction

Almost half of all patients undergoing coronary angiography for the investigation of angina have evidence of ischaemia with nonobstructive coronary arteries (INOCA) [1–3]. In the majority of cases, INOCA is caused by coronary microvascular dysfunction (CMD). CMD is more common in women and confers a higher risk of major adverse cardiovascular events. The diagnosis of CMD is supported by invasive measurement of coronary blood flow and microvascular resistance (MVR), but this is rarely performed in routine clinical practice [4]; thus, CMD remains underdiagnosed. Patients with CMD are frequently misdiagnosed with noncardiac chest pain, falsely reassured, and not provided with evidence-basedguideline-indicated treatment. This results in increased rehospitalizations, unnecessary patient morbidity, reduced quality of life, and excessive healthcare costs. Although myocardial blood flow (MBF) can be quantified noninvasively on stress perfusion CMR or positron emission tomography (PET), neither technique is routinely used in clinical practice due to practical and technical constraints; there is therefore a need for a simple noninvasive method to quantify MBF and MVR to support the diagnosis of CMD and enable stratified medical therapy.

Cardiovascular magnetic resonance (CMR) imaging techniques have advanced considerably, in recent years, in particular with the development of 4D flow imaging and quantitative myocardial perfusion [5, 6]. Quantitative perfusion can provide an estimate of MBF using time-intensity curves and kinetic modeling techniques. However, the accuracy of these methods relies on a number of assumptions. Moreover, their use in routine clinical practice is limited by extensive and complex postprocessing requirements. Quantification of coronary sinus (CS) flow may provide a simpler, more direct, and accessible method of assessing MBF. The coronary sinus receives >95% of the coronary blood flow supplied to the left ventricle and therefore provides a good representation of global left ventricular perfusion [7]. Moreover, the coronary sinus is easily imaged on CMR and, unlike the coronary arteries themselves, is large enough for accurate flow quantification. This has previously been described using two-dimensional (2D) phase contrast imaging, but these techniques have not gained favour due mainly to large exclusion rates and the extensive preacquisition planning required. 4D flow imaging techniques are emerging as the reference method for transvalvular flow assessment and may offer complementary information in routine clinical practice, providing an opportunity for quick, simple, and accessible assessment of coronary blood flow, which may yield useful information regarding coronary physiology. Whether CS flow can be quantified using 4D flow methods remains unknown.

In this study, we sought to determine whether MBF and MVR can be quantified from CS flow using 4D flow CMR imaging without the need for any additional preacquisition planning or contrast administration. We hypothesized that this technique could be utilized to detect differences in resting myocardial blood flow between patients with a history of recent MI and healthy volunteers.

## 2. Materials and Methods

### 2.1. Study Design and Population

This was an observational cohort study performed in the Department of Cardiology at Leeds Teaching Hospitals NHS Foundation Trust. Informed consent was obtained from all participating patients and healthy volunteers. The study was performed in line with the principles of the Declaration of Helsinki. Approval was granted by the local ethics committee (12/YH/0169). The control group comprised ten healthy volunteers with no previous history of cardiac disease. Patients presenting with a first-timeST-elevation myocardial infarction (MI) treated by the primary percutaneous coronary intervention (PPCI) were recruited. Patients were excluded if they had previous MI or coronary artery bypass grafting, known cardiomyopathy, an estimated glomerular filtration rate <30 ml/min/1.73 m^2^, haemodynamic instability (requiring ongoing intravenous therapy or respiratory support), or contraindication to CMR imaging. All patients underwent CMR imaging within 72 hours of the index presentation. Patients were stratified by the presence of microvascular obstruction (MVO) on CMR. Twenty patients were studied: ten with confirmed microvascular obstruction (MVO) and ten without.

### 2.2. CMR Image Acquisition

CMR images were acquired with a 1.5 Tesla CMR scanner (Ingenia, Phillips, Best, and NL), with a 28-channel flexible torso coil at the University of Leeds. The CMR protocol included a baseline survey, cine imaging (vertical long axis (2 chamber view), horizontal long axis (4 chamber view), and short axis contiguous left ventricular volume stack) acquired using balanced steady-state-free precession in a single slice breath-hold sequence, whole heart 4D flow acquired using a fast field echo pulse sequence (echo-planar imaging (EPI) with sensitivity encoding (SENSE) acceleration) with retrospective ECG triggering and 2D late gadolinium enhancement (LGE) imaging. Typical 4D flow image parameters were as follows: echo time (TE) of 3.5 s, repetition time (TR) = 10 ms, flip angle of 10 degrees, the FOV 400 × 400 mm, 30 cardiac phases, 40 slices, and VENC of 150 cm/s. The 4D flow sequence has been described in our previous papers in detail [8–11]. Average acquisition time for this technique is eight minutes per case [12]. For LGE imaging, an intravenous bolus of 0.2 mmol/kg Magnevist was administered.

### 2.3. CMR Image Analysis

**Figure 1:**
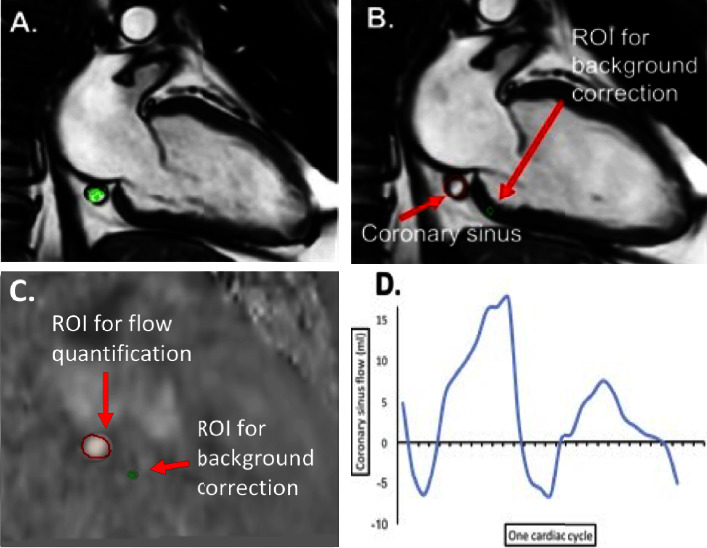
Method of coronary sinus flow quantification. The 4D flow data are loaded alongside the two-chamber view where the coronary sinus can be visualised. Alignment is checked by visualising the through-plane 4D flow at the CS using the SSFP images (a). The CS is contoured in all images. A second ROI, on the nearby myocardium, is contoured for background velocity correction (b). A reformatted phase contrast plane is generated through the coronary sinus (c) and contours are copied. Flow was measured over the entire cardiac cycle (d). *CS* *=* *coronary sinus*; *ROI* *=* *region of interest.*

Image analysis was performed offline using MASS software (version 2018EXP, Leiden University Medical Centre, Leiden, the Netherlands). Manual tracing of the epicardial and endocardial borders of the LV on short-axis cine images was performed, while excluding the papillary muscles to measure LV volumes, mass, and ejection fraction (EF). The methods for computing coronary sinus flow were adapted from those described using 2D phase contrast imaging [13]. As whole heart 4D flow data were acquired, there is no requirement for a preacquisition plane selection. The cross-sectional data routinely acquired is sufficient to allow the reformatted plane to be generated offline after the procedure. First, the contours of the coronary sinus were traced manually in the two-chamber (VLA) view, which provides a cross-sectional view of the CS. 4D flow alignment was checked by visualisation of the through-plane flow streamline. A second region of interest (ROI) on the adjacent myocardium was contoured to perform background velocity correction, as previously described [13]. A reformatted phase contrast plane was generated through the coronary sinus. CS flow was quantified over the entire cardiac cycle (Figure 1) and normalised for myocardial mass. Myocardial blood flow (MBF) was considered equal to CS flow, and where MAP is the mean systemic arterial pressure and RAP is the right atrial pressure, myocardial resistance (MyoR) was calculated according to the hydraulic equivalent of Ohm's law:

(1) $$\begin{array}{c} MyoR=\frac{\left(MAP-RAP\right)}{MBF}\text{.} \end{array}$$

RAP was estimated using the standard approach from the RA-volume analysis [8]. The RA was contoured in the 4Ch stack to provide volumetric analysis. Patients were dichotomised into those with normal and elevated right atrial pressures based on the RA end systolic volume (< or >42 ml). For the purpose of MyoR calculation, a normal RAP was assigned a value of 5 mmg and an elevated RAP of 15 mmg. MAP was obtained from blood pressure recorded at the time of the CMR scan (MAP = (systolic blood pressure + 2 (diastolic blood pressure))/3). These data were not available for healthy volunteers, so a MAP of 90 mmHg was applied to this cohort.

### 2.4. Assessment of Intra- and Interobserver Variability

To assess inter- and intraobserver variability, ten randomly selected cases were reprocessed by the same operator (RG) and by a second operator who was blinded to the initial result (GW).

### 2.5. Statistical Analysis

Continuous variables are expressed as mean (±SD) and categorical variables as number (%) unless stated otherwise. For analysis, patients were stratified into two groups depending on the presence/absence of MVO. Healthy volunteers served as the control group. Comparison of baseline characteristics between the three groups was carried out using one-way ANOVA for continuous variables and the chi-squared test for categorical variables. Comparison of CS-flow and MyoR was carried out using an independent *t* test and one-way ANOVA. To assess repeatability, Pearson's correlation coefficient and the intraclass correlation coefficient were calculated. Bland–Altman plots were created to assess agreeability between repeated measures. All statistical analyses were performed using SPSS version 26 (IBM, SPSS Inc. NY, US).

## 3. Results

### 3.1. Baseline Characteristics

The baseline characteristics of the twenty patients and the healthy controls are presented in Table 1. Patients were more likely to be smokers, overweight, male, and have a family history of coronary disease.

### 3.2. Cardiac MRI Results

Mean left ventricular end-diastolic volume (LVEDV) was 156 mm (±13.5), mean ejection fraction (EF) was 52% (±13.5), and mean LV mass was 109 g (±32.6). CMR results stratified by clinical group are shown in Table 2. Left ventricular end-diastolic volume, end-systolic volume, and mass were all greater in patients than in controls, whereas stroke volume and ejection fraction were lower in patients.

### 3.3. Coronary Sinus Flow Quantification

**Figure 2:**
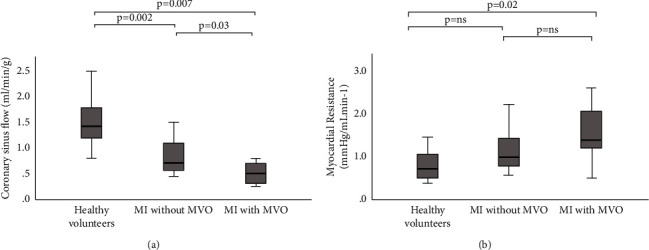
Coronary sinus flow stratified by disease subgroup. Coronary sinus flow (ml/min/g) (a) and myocardial resistance (b) in healthy volunteers, MI without MVO and MI with MVO. Data shown as boxplots and whiskers indicating the minimum and maximum, and the boxes the 25^th^ and 75^th^ percentiles. *MI* *=* *myocardial infarction*; *MVO* *=* *microvascular obstruction.*

CS flow was computed successfully in all 30 patients. Mean CS flow was 92.5 (±42.9) mL/min. When normalised for myocardial mass, the mean CS flow was 0.93 (±0.54) mL/min/g. CS flow was highest in the healthy volunteers and was significantly reduced in patients with MI and no MVO, and even lower in those with MI and MVO (123.8 (±48.4) mL/min versus 85.7 (±30.5) mL/min versus 67.9 (±29.2) mL/min, *P*=0.007) (Table 3). These differences remained significant after normalising for LV mass (Table 3 and Figure 2). Peak CS flow velocity was also significantly higher in the healthy control group compared to the two patient groups (22.7 cm/s (±6.3) versus 13.1 (±5.8) versus 15.8 (±5.1), *P*=0.003). There were no significant differences in flow or flow velocity between patients with and without MVO (*P*=0.87). MyoR was lowest in healthy volunteers and was significantly increased in patients with MI, and even higher in those with MI and MVO (0.79 (±0.35) mmHg·mL·min^−1^ versus 1.10 (±0.50) versus 1.50 (±0.69), *P*=0.02).

### 3.4. Intraobserver Variability

**Figure 3:**
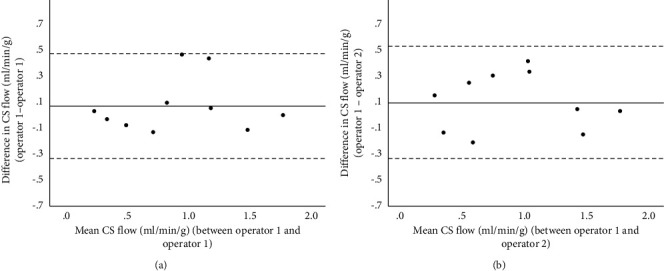
Bland–Altman plots demonstrating intra- (a) and inter- (b) observer repeatability. The dark line represents the mean delta (bias) and the dotted horizontal lines represent the limits of agreement ± 1.96 SD above and below the mean.

Between repeated results, there was a strong correlation (*r* = 0.92, *P* < 0.001) and intraclass correlation coefficient (0.92, 95% CI 0.72 to 0.98). By Bland–Altman analysis, mean bias was 0.05 (0.20) (Figure 3(a)).

### 3.5. Interobserver Variability

Between repeated results, there was a strong correlation (*R* = 0.90, *P* < 0.001) and intraclass correlation coefficient (0.90, 95% CI 0.78 to 0.98). By Bland–Altman analysis, the mean bias was 0.08 (0.22) (Figure 3(b)).

## 4. Discussion

We have quantified CS flow in ten healthy individuals and twenty patients with previous MI using whole-heart 4D flow CMR. No preacquisition planning was required and results were highly reproducible. Baseline CS flow was reduced significantly in patients with MI compared to healthy controls, with the lowest values seen in patients with confirmed MVO. Conversely, calculated MyoR was increased significantly in patients with MI, with the highest values seen in patients with confirmed MVO. Peak CS velocity was also significantly higher in the healthy control group.

More than 95% of total left ventricular coronary blood flow drains via the CS. Measurement of CS flow, therefore, provides a good estimate of left ventricular myocardial blood flow [7]. The invasive measurement of CS flow was first described in 1971 [14]. In patients with normal coronary arteries, mean resting CS flow was 122 mL/min. This is in keeping with our findings of a mean of 123 mL/min in our healthy volunteers. More recently, CS flow has been quantified using 2D phase contrast CMR [15]. Most studies have focused on the use of CS flow quantification for the diagnosis of epicardial CAD, but this has failed to gain widespread clinical adoption partly due to the additional preacquisition planning required, and partly because CS flow cannot regionalise changes in blood flow. Moreover, many studies have demonstrated high exclusion rates due to incomplete or suboptimal images, raising questions about its suitability for routine clinical use [16, 17]. Our study is the first description of CS flow quantification using 4D flow CMR. The main advantage of this approach is the lack of preacquisition planning and the ability to retrospectively analyse the CS flow from any whole-heart 4D flow sequence. Importantly, in our study, CS flow quantification was performed successfully on all 30 cases with no exclusions and minimal inter and intraobserver variability.

CS flow quantification alone cannot distinguish between epicardial and microvascular disease, but in the context of no obstructive (epicardial) coronary disease, a reduction in flow may be associated with the presence of CMD. Quantification of CS flow may, therefore, be a useful investigation for patients with INOCA. In the cardiac catheter laboratory, invasive assessment with an intracoronary pressure wire to measure fractional flow reserve (FFR), is the reference-standard assessment of epicardial lesion significance [18]. In those with unobstructed coronary arteries (FFR >0.80 or <50% stenosis on angiography), the ESC guidelines recommend (2A) further intracoronary wire-based assessment with either Doppler flow velocity measurement or thermodilution-derived mean transit time, used to derive coronary flow reserve (CFR) and either the index of microvascular resistance (IMR) using thermodilution or the hyperemic microvascular resistance (HMR) if derived from Doppler flow velocity [19]. Invasive techniques to derive absolute measurements of blood flow and resistance have also been developed [20, 21], and in the context of unobstructed coronary arteries, these additional tests of coronary blood flow are performed in the left coronary artery. The combination of a normal FFR with a reduced CFR and increased microvascular resistance is diagnostic of CMD. This is rarely performed in routine clinical practice due to the additional hardware, expertise, time, and cost of invasive assessment. There is, therefore, an unmet need for a reliable and reproducible, noninvasive method to diagnose CMD. The methods described in this pilot study have the potential to meet this need. Because CS flow is well matched with LV myocardial flow, in the absence of epicardial disease, flow reduction is likely to reflect CMD. If measured under resting and pharmacologically induced hyperaemic conditions (as is commonly used during CMR), these same methods can be used to calculate CFR. Moreover, if RAP and MAP can be measured, these same measurements can be used to determine the total myocardial resistance, as demonstrated in this study. In the absence of epicardial coronary disease, this will reflect MVR. Thus, with minimal development, the noninvasive methods described in this study could generate similar coronary physiological parameters that currently require advanced invasive testing. 4D flow CMR may, therefore, be a useful diagnostic test in patients with INOCA, or at least be an effective gatekeeper for more invasive testing. Moreover, this technique may have use in detecting abnormal blood flow in other cardiovascular conditions associated with coronary microvascular dysfunction such as hypertrophic cardiomyopathy and heart failure with preserved ejection fraction. Furthermore, work is determined to fully elucidate the potential role in these settings.

Myocardial blood flow can also be quantified on stress-perfusion imaging. Perfusion is assessed after the administration of intravenous contrast (gadolinium). Well-perfused myocardium has a shorter T1 and, therefore, appears brighter allowing visual detection of perfusion defects. For quantitative assessment, rapid repeat acquisitions are performed at multiple short-axis locations. The measured arterial input function and tissue signal intensity are then used to construct time-intensity curves, and kinetic modeling is applied to quantify MBF. This relies on assumptions regarding the distribution kinetics of contrast agents and the relationship between contrast agent concentration and signal intensity. For example, gadolinium concentration is only linear with signal intensity at lower concentrations; at higher levels signal saturation can lead to inaccuracies. The presence of artefacts such as dark rim artefacts can also impede accuracy. However, perhaps the biggest limitation is the lengthy and costly postprocessing required, which means it is not widely used or available in routine clinical practice. Quantifying MBF from CS flow is more direct, requires fewer assumptions, and avoids the need for contrast administration and prohibitively complex imaging protocols.

## 5. Limitations

The sample size in this pilot study was modest, but despite this, we did demonstrate statistically significant differences between all three groups. Perhaps the main limitation of this study was that measurements were acquired only during resting conditions. It would be relatively simple but highly advantageous to also acquire measurements with pharmacologically induced hyperemia so that CFR could be additionally measured. This will be tested in a future study. Our CS method cannot identify regional perfusion defects. However, the clinical rationale for this study was for patients with INOCA, in whom a global measurement is more useful. The small size of the CS requires careful alignment for 4D flow simulation to ensure accuracy. In this study, results were highly reproducible both on an intra- and interobserver analysis. However, we did not assess scan-scan variability relating to slice positioning, which will need to be addressed in a future study.

## 6. Conclusion

Myocardial blood flow and myocardial resistance can be quantified using 4D-flow cardiac MR imaging of the coronary sinus. No preacquisition planning was required, and results were highly reproducible. The technique detected significant differences in myocardial blood flow between patients with myocardial infarction and healthy volunteers. This quick, simple, and widely accessible technique may be valuable in the assessment of patients with INOCA and in the diagnosis of coronary microvascular dysfunction.

## Figures and Tables

**Table 1:** Baseline characteristics stratified by clinical group.

	Healthy controls (*n* = 10)	MI without MVO (*n* = 10)	MI with MVO (*n* = 10)	*P* value
Age	53 (±4.1)	53 (±3.3)	54 (±8.8)	0.98
BMI	26.1 (±2.6)	25.1 (±4.6)	30.0 (±4.6)	0.03
Sex (male)	4 (40%)	8 (80%)	9 (90%)	0.04
Current smoker	0 (0%)	7 (70%)	5 (50%)	0.004
Hypertension	0 (0%)	1 (10%)	1 (10%)	0.59
FH of CAD	0 (0%)	5 (50%)	4 (40%)	0.04

BMI = body mass index; FH = family history; MI = myocardial infarction; MVO = microvascular obstruction.

**Table 2:** Cardiac MRI results stratified by clinical group.

	Healthy volunteers (*n* = 10)	MI without MVO (*n* = 10)	MI with MVO (*n* = 10)	*P* value
LVEDV	131 (±24.0)	148 (±22.8)	188 (±47.2)	0.002
LVESV	46.9 (±12.4)	71 (±20.9)	118 (±44.4)	<0.001
Stroke volume	84.4 (±13.6)	77.5 (±10.4)	69.1 (±16.2)	0.06
LVEF (%)	65 (±5.2)	52 (±7.4)	38 (±9.9)	<0.001
LV mass	82 (±14.6)	110 (±22.5)	134 (±34.3)	<0.001

LVEDV = left ventricular end diastolic volume; LVESV = left ventricular end systolic volume; LVEF = left ventricular ejection fraction; MI = myocardial infarction; MVO = microvascular obstruction.

**Table 3:** Coronary sinus flow results stratified by clinical group.

	Healthy volunteers (*n* = 10)	MI without MVO (*n* = 10)	MI with MVO (*n* = 10)	*P* value
CS flow (mL/min)	123.8 (±48.4)	85.7 (±30.5)	67.9 (±29.2)	0.007
CS flow (mL/min/g)	1.48 (±0.47)	0.81 (±0.35)	0.51 (±0.19)	<0.001
Peak CS velocity (cm/s)	22.7 (±6.3)	13.1 (±5.8)	15.8 (±5.1)	0.003
MyoR (mmHg·min·mL^−1^)	0.79 (±0.35)	1.10 (±0.50)	1.50 (±0.69)	0.02

CS = coronary sinus; MI = myocardial infarction; MVO = microvascular obstruction; MyoR = myocardial resistance.

## Data Availability

Data are made available upon reasonable request to the corresponding author.

## Abbreviations

2D: Two-dimensional
4D: Four-dimensional
BMI: Body mass index
CFR: Coronary flow reserve
CMD: Coronary microvascular dysfunction
CMR: Cardiac magnetic resonance
CS: Coronary sinus
EF: Ejection fraction
EPI: Echo-planar imaging
FFR: Fractional flow reserve
IMR: Index of microvascular resistance
INOCA: Ischaemia with nonobstructed coronary arteries
LVEDV: Left ventricular end diastolic volume
LVEF: Left ventricular ejection fraction
LVESV: Left ventricular end systolic volume
MAP: Mean arterial pressure
MBF: Myocardial blood flow
MI: Myocardial Infarction
MVO: Microvascular obstruction
MVR: Microvascular resistance
MyoR: Myocardial resistance
RAP: Right atrial pressure
ROI: Region of interest.
